# An esophagus-sparing technique to limit radiation esophagitis in locally advanced non-small cell lung cancer treated by simultaneous integrated boost intensity-modulated radiotherapy and concurrent chemotherapy

**DOI:** 10.1186/s13014-018-1073-3

**Published:** 2018-07-17

**Authors:** Li Ma, Bo Qiu, QiWen Li, Li Chen, Bin Wang, YongHong Hu, MengZhong Liu, Li Zhang, Yan Huang, XiaoWu Deng, YunFei Xia, MaoSheng Lin, Hui Liu

**Affiliations:** 1 State Key Laboratory of Oncology in South China, Sun Yat-sen University Cancer Center, Guangzhou, Guangdong 510060 People’s Republic of China; 2 Collaborative Innovation Center for Cancer Medicine, Sun Yat-sen University Cancer Center, Guangzhou, Guangdong 510060 People’s Republic of China; 3 Department of Radiation Oncology, Sun Yat-sen University Cancer Center, 651 Dongfeng Road East, Guangzhou, Guangdong 510060 People’s Republic of China; 4 Department of Medical Oncology, Sun Yat-sen University Cancer Center, Guangzhou, Guangdong 510060 People’s Republic of China

**Keywords:** Non-small cell lung cancer, Chemoradiotherapy, Esophagus-sparing technique, Esophagitis, Loco-regional failure free survival

## Abstract

**Background:**

To investigate the incidence of radiation esophagitis (RE) and tumor local control using esophagus sparing technique in locally advanced non-small cell lung cancer (LANSCLC) treated by simultaneous integrated boost intensity-modulated radiation therapy (SIB-IMRT) and concurrent chemotherapy.

**Methods:**

Eighty-seven patients with stage IIIA/B NSCLC who received definitive SIB-IMRT and concurrent chemotherapy had been divided into two groups: 1.with esophagus sparing technique; 2.without esophagus sparing technique. Chi-square test was performed to compare sex, clinical stage, histology, concurrent chemotherapy, RE and nutrition status between two groups. T-test was used to compare the dosimetric parameters. Overall survival (OS) and loco-regional failure free survival (LRFS) were calculated by the Kaplan–Meier method and compared by a log-rank test.

**Results:**

There were 44 patients in the esophagus sparing group and 43 in the non-sparing group. The incidence of severe RE (Grade 3) was significantly lower in patients with esophagus sparing technique (*p* = 0.002). Patients in esophagus sparing group had better nutrition status (*p* = 0.045). With a median follow-up of 18 months (range 1–51 months), the 1-year, 2-year and 3-year OS of all the patients was 86.6, 65.4 and 43.7%. The 1-year, 2-year LRFS was 78.4, 65.9%. OS time (*p* = 0.301) and LRFS (*p* = 0.871) was comparable between two groups.

**Conclusions:**

Esophagus-sparing technique is an effective and essential method to limit RE in LANSCLC treated by SIB-IMRT and concurrent chemotherapy without compromising local control.

## Background

High-dose radiotherapy with concurrent chemotherapy is a radical treatment option in stage III non-small cell lung cancer (NSCLC) patients who are not suitable for surgery. The esophagus is a critical organ for dose constraints in definitive radiotherapy of lung cancer [1, 2]. The incidence of severe acute esophagitis (grade 3 or higher) was reported to be about 8.7–20% in NSCLC patients treated with IMRT-based concurrent chemoradiotherapy [3, 4]. Severe acute esophagitis usually results in poor nutrition status, interruption of radiotherapy, and low tolerance of concurrent chemotherapy, which might have adverse effects on long term treatment outcome [5–7].

Extensive studies explored the predictors of grade 3 or higher radiation-related esophagitis. Most results showed that the severity of esophagitis was significantly related to dosimetric factors. V50 (volume of esophagus receiving ≥50 Gy) and V60 were reported most to have significant associations with severe esophagitis in patients treated with chemoradiation [2, 8]. Recent studies showed that concurrent chemotherapy and hyperfrationated radiotherapy improved overall survival and local control; however, higher incidence of severe esophagitis had been observed in patients using these treatment schemes [9–14].

Results of RTOG 0617 showed that mean lung dose, lung V20, esophagus dose and heart dose were significantly higher in patients who received high-dose chemoradiotherapy than those in standard-dose group, and treatment-related deaths were more common in the high-dose group. In multivariate analysis,heart V5 and V30 were found to be related with patient death. Greater toxicity accompanied with dose escalation might contribute to the differences in survival [15]. Thus, limiting the exposure of dose-limiting critical organ at risk (OAR) needs to be taken into account in NSCLC patients who receive concurrent chemoradiotherapy.

Previous results suggested that it may be feasible to deliver simultaneously a higher dose per fraction to the primary disease and a relatively lower dose to the subclinical or selected other regions by using simultaneous integrated boost intensity-modulated radiation therapy (SIB-IMRT) [16, 17]. Some results showed that a relatively longer median survival time for locally advanced Stage III NSCLC could be achieved by SIB-IMRT. The relative good loco-regional failure free survival (LRFS) might benefit from the higher biological equivalent dose (BED) of radiation [18]. However, to constraint dose to OARs strictly might be the precondition to increase the radiation dose safely.

In our study, SIB-IMRT-based esophagus sparing technique was used to increase daily fraction size to the gross tumor volume, while limiting esophagus irradiation dose. We aimed at comparing the incidence of radiation esophagitis and tumor control between patients treated with or without esophagus sparing technique.

## Methods

### Acquisition of clinical data

We retrospectively reviewed the records of 87 consecutive patients diagnosed and pathologically confirmed IIIA-IIIB NSCLC, treated by SIB-IMRT technique and concurrent chemotherapy from January 2012 to December 2014 in the Sun Yat-sen University Cancer Center. SIB-IMRT has been utilized to treat locally advanced NSCLC in our center since 2012. At the beginning, the esophagus was not strictly spared. The max dose of esophagus was constrained < 105% of prescription dose and the mean dose <= 34Gy according to NCCN guideline. However, the incidence of G3–4 esophagitis during and after SIB-IMRT CCRT significantly increased, which prolonged the in-hospital time and impaired the quality of life. The reason could be Asian patients had the trend to develop severe esophagitis, and our previous study suggested that malnutrition caused by prolonged esophagitis was associated with the risk of severe radiation pneumonitis [19]. In order to decrease the esophagitis, we have been using esophagus sparing technique to strictly constrain the irradiated dose of esophagus since 2013. In this study, the 44 patients in the sparing group were treated from 2013 to 2014, and the 43 patients in non-sparing group were mostly treated prior to 2013. Dosimetric parameters were recorded from dose-volume histogram (DVH), including mean dose, max dose, V45, V50, V55, V60 of esophagus. The 7th edition of American Joint Committee on Cancer (AJCC) staging system for lung cancer was used to stage the diseases. The routine staging process included a complete medical history and clinical examination of the head and neck region, bronchoscopy, CT of the chest and upper abdominal, magnetic resonance imaging (MRI) of the brain and chest, a whole-body bone scan, or positron emission tomography (PET)-CT. Written informed consent was obtained from the patient for the publication of this report.

### Radiotherapy

**Fig. 1:**
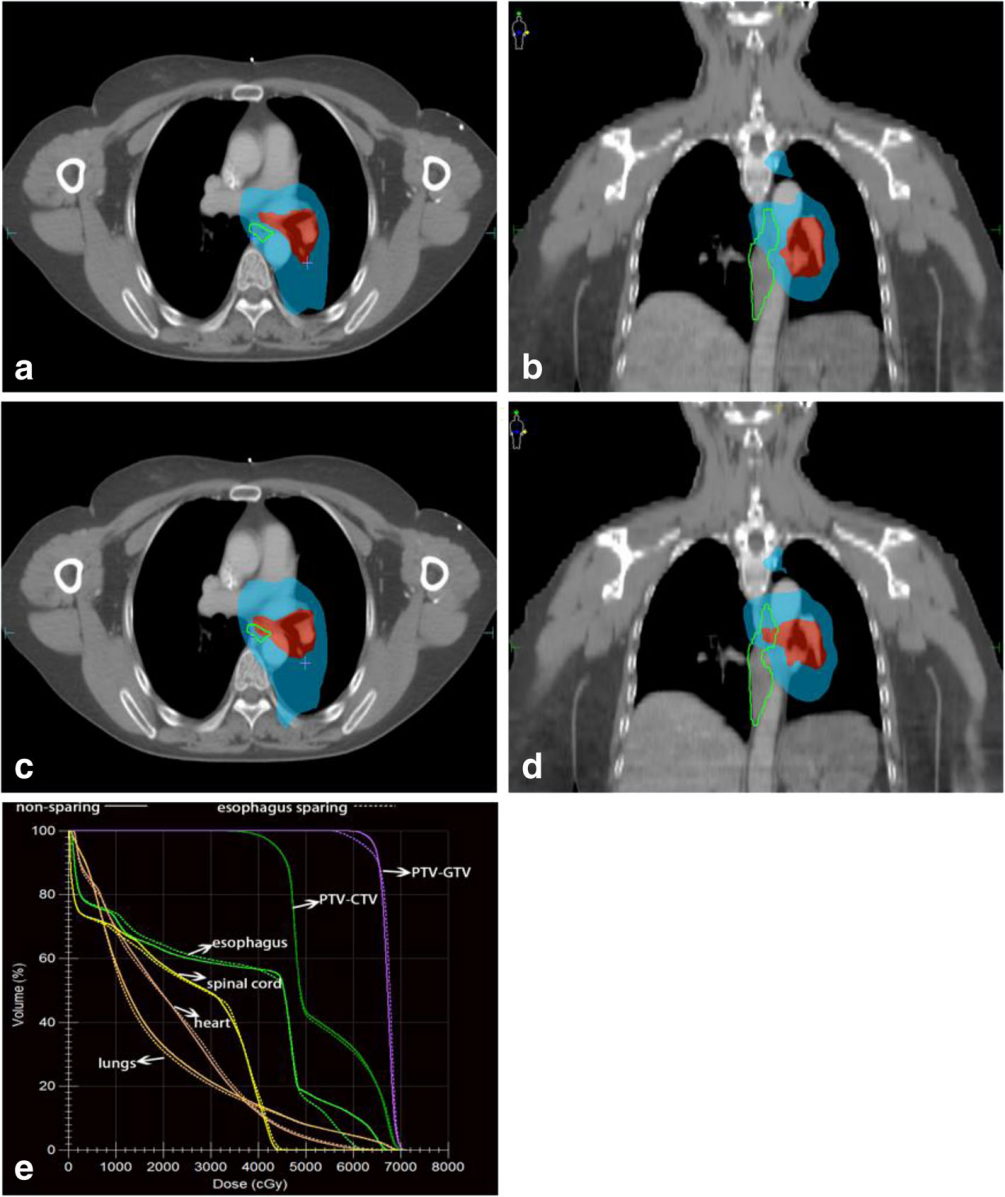
**a**, **b** show the dose distribution of one patient using esophagus-sparing technique, and **c**, **d** without esophagus-sparing technique. Doses were delivered using simultaneous integrated boost intensity modulated radiotherapy with 65Gy (red area) to the PTV-GTV and 45Gy (blue area) to the PTV-CTV. **e** a dose-volume histogram comparing the two plans

SIB-IMRT and esophagus sparing SIB-IMRT was planned for all patients (Fig. 1). Patient immobilization, simulation and treatment planning were performed according to standard protocol for lung cancer receiving radiotherapy in our department. With the patient in supine position, a cradle for immobilization was made with vacuum. A treatment planning CT scan was performed with 0.5 cm thickness slices from the Atlas (C1) to the second lumbar vertebra (L2) level to cover the whole neck and lung. The 4DCT scanning was performed and the respiration motion of patients was recorded. The images were sorted into 10 phases representative of a single respiratory cycle. The maximum intensity projection (MIP) images of 4DCT were also reconstructed. Briefly, Gross tumor volume including all known tumor and mediastinal lymph node was defined on the each phase of the 4D planning CT. The internal target volume (ITV) was defined as the composite volumes of CTVs across the 10 phases of the breathing cycle referring to MIP images. Gross tumor volume was defined as any visible primary lesions by CT/PET-CT/MRI scans, and all lymph nodes with a diameter ≥ 1 cm in short axis or standard uptake value (SUV) ≥2.5 were included. Clinical target volume was defined as the high-risk lymph nodal regions, including adjacent regions of involved lymph nodes (e.g. 2 left (L), 5, and 7 would be included in the clinical target volume if 4 L was involved), and the ipsilateral hilar in accordance with the new lymph node map of the International Association for the Study of Lung Cancer, including the gross tumor volume with a 0.5–0.8 cm margin. Another 0.5 cm margin was added to create the planning target volume (PTV). The PTV-GTV was formed by including a 0.5 cm margin around the GTV and the PTV-CTV was formed by including a 0.5 cm margin around the CTV. The median prescribed dose to PTV-GTV was 65Gy (range from 60Gy to 67.6 Gy), median fraction size 2.5Gy (range from 2.2Gy to 3.2Gy). The dose to PTV-CTV ranged from 45 to 50Gy. Dose constraint for critical organs include: the maximum spinal cord dose < 46 Gy, mean lung dose ≤17 Gy and V20 ≤ 35%, V40 of heart ≤40%.

The length of the esophagus was defined to extend from the inferior border of the cricoid cartilage to the gastro-esophageal junction. The external surface of esophagus was contoured on each axial slice of the CT images used for treatment planning. Patients had been divided into two groups according to esophagus dose constraint retrospectively (Fig. 1). Group 1: SIB-IMRT with esophagus sparing; Group 2: SIB-IMRT without esophagus sparing. The details of esophagus sparing technique included: 1. a margin of 3 mm had been added to the esophagus to allow dose decline (eg. from 65 Gy prescription dose to 60Gy at the edge of esophagus); 2. maximum dose to esophagus was restricted to < 65 Gy and V50 < 30%; 3.minimum dose to PTV-GTV was required as > 60 Gy [15]; 4. Image guidance with daily cone beam CT (CBCT) was performed for positioning assurance. For patients without esophagus sparing, the max dose of esophagus was constrained < 105% of prescription dose and the mean dose <= 34Gy according to NCCN guideline.

### Concomitant chemotherapy

Most patients in this study received weekly paclitaxel/ docetaxel (25 mg/m^2^) and cisplatin/nedaplatin (25 mg/m^2^) concurrent with radiotherapy. Pemetrexed (500 mg/m^2^) and cisplatin/ nedaplatin (75 mg/m^2^) every 3 weeks was also used.

### Toxicities and follow-up

Overall survival (OS) was defined as the time from the initial date of radiotherapy to the date of death from any cause or to the last visit before May 31, 2016, which was censored at the date of last follow up. LRFS was calculated from the initial date of radiotherapy to the date of loco-regional progression or the date of last visit before May 31, 2016. The primary clinical endpoint was LRFS. OS and treatment related toxicities were also recorded. Radiation oncologists evaluated patients weekly during treatment, and then every 3–6 months after concurrent chemoradiotherapy (CCRT) for at least 2 year follow up. Chest and upper abdomen scan CT were performed at each follow-up evaluation after completion of treatment. Any suspect recurrence should be confirmed by endoscopy. Brain magnetic resonance imaging (MRI) was performed each 6 months. Bone scan was administrated when patients were suspected for bone metastasis. The maximum RE grade at each patient and Subjective Global Assessment (SGA) score were recorded. Acute esophagitis was assessed by Common Terminology Criteria for Adverse Events Version 4.0 (CTCAE 4.0) from the start of radiotherapy until 3 months afterward. Patients were evaluated through the SGA before and at the end of treatment. Nutritional status was classified as A (well-nourished), B (suspected malnutrition or moderately mal-nourished), or C (severely mal-nourished) [20].

### Statistical analysis

Patients were divided into two groups according to the use of esophagus sparing technique. Chi-square test was performed to evaluate the risk of sex, age, clinical stage, N stage, histology, radiotherapy dose, biological effective dose (BED), GTV volume, concurrent chemotherapy, RE and nutrition status between these two groups. T-test of independent sampler was used to compare the dosimetric parameters of the two groups. OS and LRFS were calculated by the Kaplan–Meier method, respectively, and differences in survival were assessed by a log-rank analysis. All statistical analyses were performed using SPSS 22.0 software (IBM, Chicago, IL, USA).

## Results

### Patient characteristics

A total of 87 patients met the criteria for inclusion in this study. There were 44 patients in esophagus sparing group and 43 patients in non-sparing group. Patient characteristics were detailed in Table 1. Thirty-seven (42.5%) and 50 (57.5%) patients had stage IIIA and IIIB disease at initial diagnosis, respectively. The median minimum distance between GTV and esophagus was 7 mm (0-32 mm). The median BED (The alpha/beta ratio of tumor was defined as 10) was 81 Gy (range, 71-85Gy). Weekly nedaplatin/cisplatin and paclitaxel/docetaxol were the most commonly used agents for concurrent chemotherapy (*n* = 72, 82.8%), while nedaplatin/cisplatin and pemetrexed accounted for 17.2% (*n* = 15).

**Table 1:** Baseline characteristics of patients

Characteristics	Patients (*n* = 87) No. (%)		*P* value
	Esophagus-sparing (*n* = 44)	Standard (*n* = 43)	
Sex			0.066
Male	32	38	
Female	12	5	
Age (year), median (range)	56 (34–76)	58 (33–77)	0.476
Clinical stage of primary tumor (7th edition)			0.577
IIIA	20	17	
IIIB	24	26	
N stage			0.120
1	4	0	
2	24	24	
3	16	19	
Histology			0.646
Adenocarcinoma	22	19	
Squamous cell carcinoma	20	20	
Other	2	4	
RT dose (Gy), median (range)	63.8 (60–66)	65 (63–67.6)	0.068
BED (Gy), median (range)	81 (74–85)	81 (78–85)	0.064
GTV volume (cm^3^), median (range)	83.7 (25.1–275.3)	88.2 (22.3–281.4)	0.472
Minimum distance between GTV and esophagus (mm)	7 (0–32)	6 (0–29)	0.621
Concurrent chemotherapy			0.422
Nedaplatin or cisplatin + Paclitaxel/docetaxel	35	37	
Nedaplatin or cisplatin + Pemetrexed	9	6	
Radiation esophagitis			0.002
G0–2	42	30	
G3	2	13	
SGA (before CCRT)			0.670
A	41	39	
B	3	4	
SGA (after CCRT)			0.045
A	36	25	
B	8	17	
C	0	1	
Hearth V30 (%)	20.70	18.20	0.665
Dose to the esophagus			
Dmax (Gy)	64.67	70.03	0.002
Dmean (Gy)	26.25	36.77	0.000
V45 (%)	33.76	54.51	0.000
V50 (%)	20.75	48.41	0.000
V55 (%)	11.25	37.41	0.000
V60 (%)	6.12	21.64	0.000

*Abbreviations: RT* Radiotherapy, *BED* Biological effective dose, *SGA* Subjective Global Assessment, *CCRT* Concurrent chemoradiotherapy, *Dmax* max dose, *Dmean* Mean dose, *Vx* Volume of esophagus receiving x Gy

### Radiation esophagitis

**Fig. 2:**
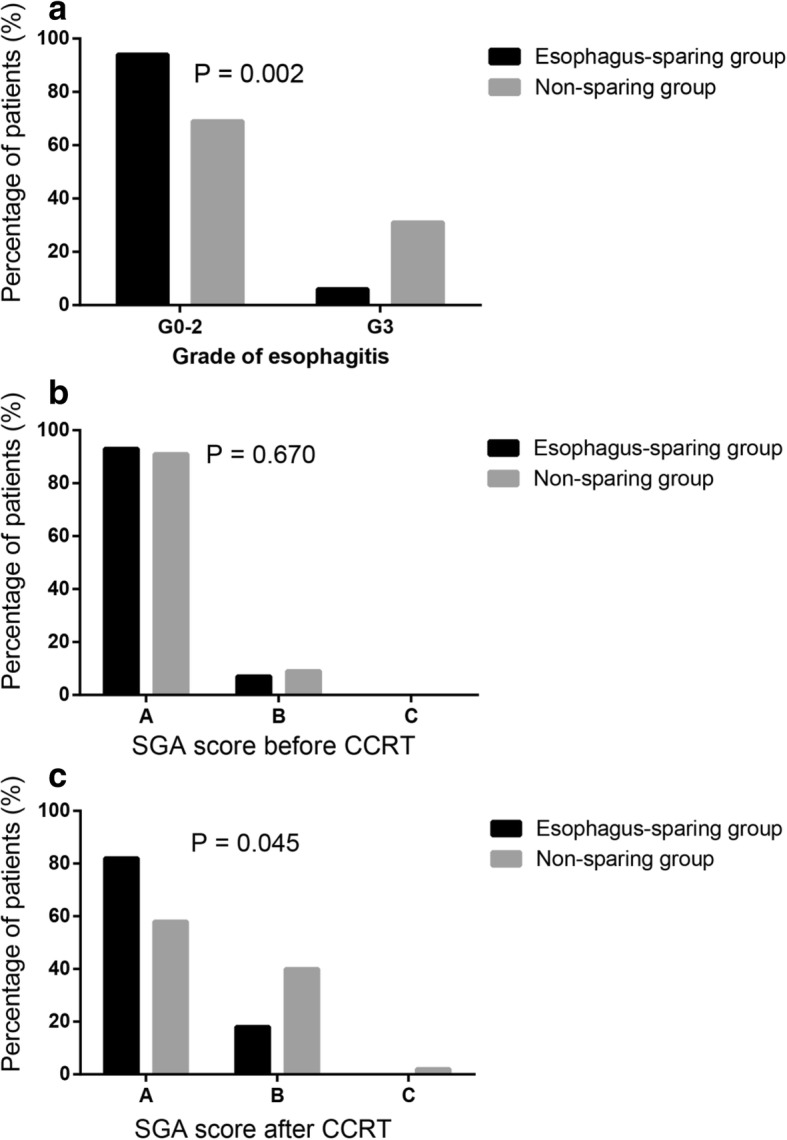
**a** Grade of esophagitis for esophagus-sparing group and non-sparing group. The grade of RE (*p* = 0.002) had statistical significance between two groups. Patients had lower incidence of RE in the esophagus sparing group. **b**, **c** represent Subjective Global Assessment (SGA) score for esophagus-sparing group and non-sparing group. **b** SGA before treatment. **c** SGA after treatment. Patients in two groups had comparable SGA scores before treatment (*p* = 0.670). However, post-treatment SGA score (p = 0.045) showed significant association with the use of esophagus sparing. Patients in esophagus-sparing group had better nutrition status

Of the 87 patients included in our study, no grade 4 or 5 RE was found. The rate of esophagitis of any grade (Grade 1–3) was 95.4% (*n* = 83), including 28.7% for Grade 1 (*n* = 25), 49.4% for Grade 2 (*n* = 43) and17.2% for Grade 3 (n = 15). In the esophagus-sparing group, Grade 1 esophagitis appears in 18 patients (40.9%), Grade 2 in 20 patients (45.5%) and Grade 3 in 2 patients (4.5%). In the non-sparing group, Grade 1 esophagitis appears in 7 patients (16.3%), Grade 2 in 23 patients (53.5%) and Grade 3 in 13 patients (30.2%). Patients had significantly lower incidence of G3 RE in the esophagus sparing group (*p* = 0.002). (Fig. 2a).

### SGA score

SGA score was evaluated before and at the end of treatment. Before treatment, 92.0% (*n* = 80) and 8.0% (n = 7) patients were assessed as A and B, respectively. At the end of treatment, 70.5% (*n* = 67), 28.4% (*n* = 27) and 1.1% (n = 1) were assessed as A, B and C, respectively. In esophagus-sparing group, 36 patients got A score, 8 got B; Without using esophagus-sparing technique, 25 patients got A score, 17 got B, 1 got C. Post-treatment SGA score (*p* = 0.029) had significant association with the use of esophagus sparing (Fig. 2b, c).

### Dosimetric parameters

Dosimetric parameters of two groups were showed in Table 1. The average mean dose, max dose, V45, V50, V55 and V60 of esophagus were significantly lower in the esophagus-sparing group.

### Follow-up

**Fig. 3:**
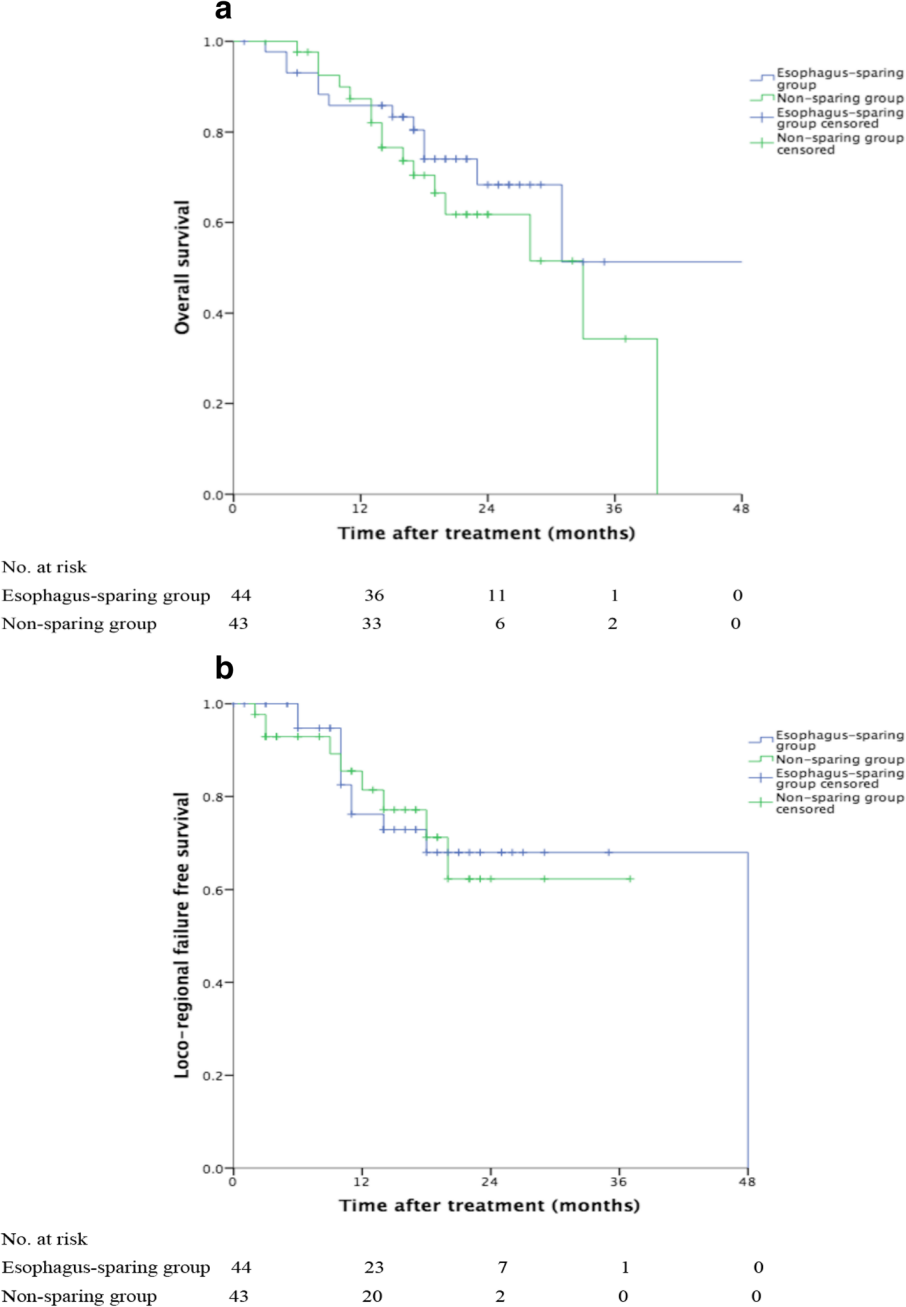
**a** Overall survival (OS) curve for esophagus-sparing group and non-sparing group. The difference between two groups was not significant (Log-rank, *P* = 301) (**b**) Loco-regional failure free survival (LRFS) curve for esophagus-sparing group and non-sparing group. The difference between two groups was not significant (Log-rank, p = 0. 871)

**Fig. 4:**
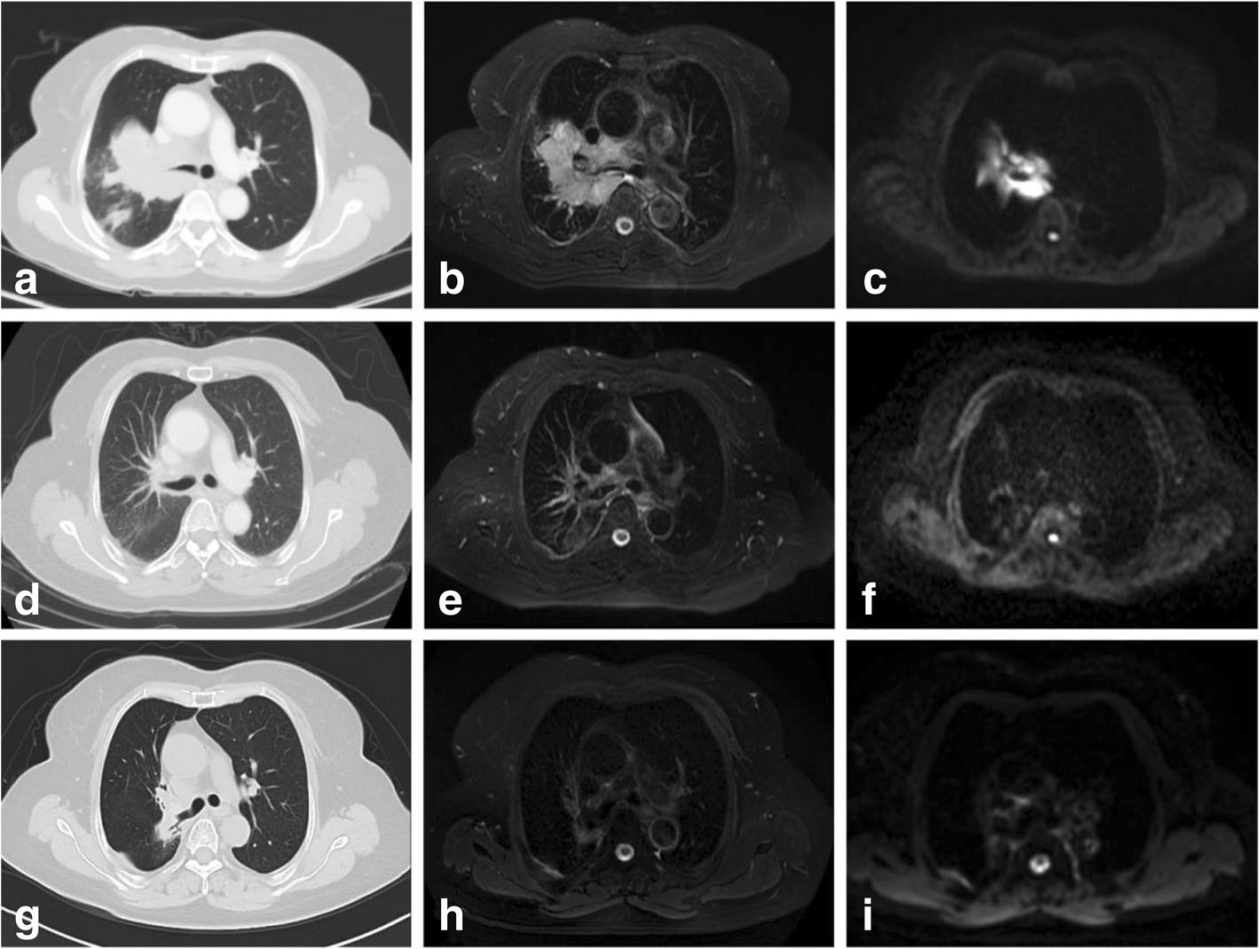
Treatment outcome of one patient in esophagus-sparing group. Before SIB-IMRT and chemotherapy, a tumor located in right hilum was revealed on, CT (**a**), T2WI (**b**) and DWI (**c**). Two months after SIB-IMRT and chemotherapy, no visible tumor was observed on CT (**d**), T2WI (**e**) and DWI (**f**). Fifteen months after SIB-IMRT the tumor was well controlled (**g**, **h**, **i**)

With a median follow-up of 18 months (range 1–51 months), the 1-year, 2-year and 3-year OS of all the patients was 86.6, 65.4 and 43.7%. the 1-year, 2-year LRFS was 78.4, 65.9%. OS time (*p* = 0.301) and LRFS (*p* = 0.871) was comparable between two groups with or without esophagus sparing (Fig. 3). Figure 4 showed the treatment outcome of one patient in esophagus-sparing group. This patient had complete remission after treatment and remained free of disease 15 months from radiotherapy.

## Discussion

Recently, OARs sparing technique based on IMRT were introduced to reduce the incidence of radiation toxicities. Contralateral esophagus-sparing technique (CEST) proposed by Al-Halabi.H et al. [21] was performed in 20 consecutive patients with a satisfied dose delivery (median radiation dose was 70.2 Gy). No patients developed grade ≥ 3 esophagitis. In another study, 82 lung cancer patients were recruited. Of them, 44 were treated with esophagus and contralateral lung-sparing radiation therapy (95% using IMRT and 5% using 3DCRT), while 38 patients received non-sparing radiation therapy (45% using IMRT and 55% using 3DCRT). The incidence of acute grade ≥ 3 esophagitis was much lower in the esophagus and contralateral lung-sparing radiation therapy group (0% vs. 11%, *p* < 0.001) [22]. In our study, the baseline characteristics, radiation dose, GTV volume and concurrent chemotherapy were balanced between these two groups (Table 1). The incidence of grade 3 RE (no grade 4 or 5 RE was found) in patients treated with esophagus sparing technique was 4.5%, which was significantly lower than the non-sparing group (30.2%, *p* = 0.002). We also found that nutritional status was statistically different between two groups (*p* = 0.045).Therefore, limiting the volume and dose of esophagus receiving radiation could help reduce the incidence of severe RE and improve patients’ nutrition status.

Esophagus-sparing was concerned to sacrifice efficacy, because the mediastinal lymph nodes and central tumor were often close to the esophagus [22]. Recently, SIB-IMRT has been used to deliver high dose to the tumor and decrease the dose of OARs simultaneously [23]. In our study, we limited high dose of esophagus and ensure PTV dose ≥60 Gy at the same time using SIB-IMRT technique. Through long-term follow-up, comparing the esophagus-sparing and non-sparing group, LRFS (*p* = 0.871) showed no statistical significance. This result suggests that with PTV dose ≥60 Gy, the esophagus-sparing technique was feasible without compromising local control. It is noteworthy that, SIB-IMRT can achieve highly conformal dose distribution with sharp dose gradients. Accordingly, it is important to routinely perform CBCT (Image guided radiation therapy, IGRT) to ensure the accurate tumor localization and dose delivery.

Kao et al. [22]showed that patients treated with normal tissue-sparing radiation therapy achieved improved survival compared to patients treated with radiotherapy without normal tissue sparing. However, the radiotherapy dose, technique and the use of chemotherapy were not equally distributed between the two groups. These intergroup differences might have contributed to the difference in survival. In our study, the baseline characteristics and  potentially prognostic of survival were comparable between two groups, including clinical stage, N stage, RT dose, BED dose and GTV volume. OS time showed no difference between the two groups, although there was a trend towards longer OS time in the sparing group.

Different from the previous research by Al-Halabi.H [21], we performed the whole esophagus-sparing technique other than contralateral esophagus sparing. It is based on the rationale that esophagus arranges in a serial fashion that the inactivation of even a single functional subunit can impact the function of the entire organ for tissues [24]. Part of esophagus irradiated with extremely high dose can cause serious complication like perforation. Therefore, we contoured the whole esophagus and limit the maximum dose in order to protect the whole esophagus instead of part of it.

In this study, we proposed a novel whole esophagus-sparing technique, and showed its feasibility without compromising efficacy. But our study still has several shortcomings. It is a retrospective study with small sample size. Although there was no significant difference in baseline characteristics between the two groups, there may be selective bias and other confounding factors.

## Conclusions

Esophagus-sparing technique is an effective and essential method to limit radiation esophagitis in locally advanced NSCLC patients treated by SIB-IMRT and concurrent chemotherapy. Reducing severe esophagitis while escalating radiation dose may help to achieve better local control and general performance status.

## Abbreviations

AJCC: American Joint Committee on Cancer
BED: Biological equralent dose
CBCT: Cone beam CT
CCRT: Concurrent chemoradiotherapy
CEST: Contralateral esophagus-sparing technique
CTCAE: Common Terminology Criteria for Adverse Events
CTV: Clinical target volume
DVH: Dose-volume histogram
GTV: Gross tumor volume
IGRT: Image guided radiation therapy
LANSCLC: Locally advanced non-small cell lung cancer
LRFS: Loco-regional failure free survival
MIP: Maximum intensity projection
MRI: Magnetic resonance imaging
OAR: Organ at risk
OS: Overall survival
PTV: Planning target volume
RE: Radiation esophagitis
SGA: Subjective Global Assessment
SIB-IMRT: Simultaneous integrated boost intensity-modulated radiation therapy
